# Irritability in Children with Rasopathies, Insights into Emotional Dysregulation and Social Skills Impairments

**DOI:** 10.21203/rs.3.rs-5428038/v1

**Published:** 2024-12-19

**Authors:** Yaffa Serur, Naomi Fuhrman, Odeya Russo, Tamar Green

**Affiliations:** Stanford University; Stanford University; Stanford University; Stanford University

**Keywords:** Rasopathies, Noonan syndrome, Neurofibromatosis type 1, Irritability, Emotional dysregulation-related disorders, Social skills impairments

## Abstract

Rasopathies, including Noonan Syndrome (NS) and Neurofibromatosis type 1 (NF1), are developmental disorders caused by germline mutations in genes of the RAS/mitogen-activated protein kinase pathway (RAS-MAPK). This study investigates irritability, a highly prevalent transdiagnostic construct, in children with Rasopathies and the impact of Rasopathy status on the associations between irritability, emotional dysregulation-related disorders, and social skills impairments.

The sample comprise 174 children aged 4–17 (age mean = 9.49; 98 females), including 113 children with Rasopathies (NS n = 85, NF1 n = 28) and 61 age-sex-matched typically developed (TD) children. We used parent questionnaires (CBCL, SRS) to assess irritability, symptoms of ADHD, defiance, anxiety/depression, and social skills impairments while controlling for cognitive measures (IQ).

Children with Rasopathies exhibited higher irritability than TD children (mean difference = 1.09; p < 0.001). Children with NS showed a weaker association between irritability and ADHD symptoms compared to TD children (*p* = .032, η_p_^2^ = .03) and a stronger association between irritability and social skills impairments compared to both TD (*p* = .033, η_p_^2^ = .03), and NF1 groups (*p* = .009, η_p_^2^ = .06).

We present novel and clinically significant findings showing high irritability in children with Rasopathies. Our study provides syndrome-specific results, suggesting differences in the mechanisms involved in irritability, ADHD, and social processes in children with NS and NF1. In essence, children with Rasopathies showed a highly irritable profile associated with ADHD symptoms and social skills impairments, with a significantly stronger association between irritability and social processes in NS. Our results suggest that developing prevention and treatments targeting irritability can distinctly affect the trajectories of neurodevelopmental disorders in children with Rasopathies.

## Introduction

The Rasopathies is a group of developmental disorders caused by germline mutations in genes encoding protein components of the RAS/mitogen-activated protein kinase (RAS-MAPK) pathway [1], which regulates cell growth processes [2]. While Rasopathies are associated with increased RAS-MAPK pathway signaling and share similar clinical features, including developmental delay, physical problems, and increased risk for malignancies, each Rasopathy is characterized by a unique and distinct phenotype [3]. The two most common Rasopathies are Noonan Syndrome (NS) and neurofibromatosis type 1 (NF1)[1].

NS is estimated to occur in approximately 1:1000–2500 live births; it is a disorder predominantly transmitted in an autosomal dominant manner and in approximately 50% of cases, the identified causative gene is the protein tyrosine phosphatase, the non-receptor type 11 (*PTPN11)* [4]. NF1 occurs in approximately 1:3000 live births; it is a disorder predominantly transmitted in an autosomal dominant manner and is caused by mutations or deletions of the *NF1* gene located on chromosome 17 [5]. Recent studies indicate that the dysregulation of the Ras-MAPK pathway may lead to similar behavioral and psychiatric comorbidities across children with NS and NF1. While studies in both NS and NF1 have found an increased prevalence of cognitive and learning disabilities, ADHD, and social skills impairments associated with ASD [18–20], transdiagnostic symptoms like irritability have not been examined, undermining our ability to understand the mechanisms involved in irritability and to detect the differences between distinct Rasopathies.

Irritability is conceptualized as a low threshold for experiencing anger in response to frustration [8]. It is a dimensional trait undermining the divide between internalizing and externalizing psychiatric diagnoses [7], and it is one of the constructs of emotional dysregulation, defined as excessive and rapidly shifting emotions inappropriate to the situational context, age, and developmental stage [6]. While irritability is conceived as a distinct construct in psychiatry, it is a central or dimensional feature of several psychiatric disorders, including oppositional defiant disorder (ODD), bipolar disorder, depression, conduct disorder, attention deficit hyperactivity disorder (ADHD), and anxiety [9–12]. Irritability in children has been associated with adverse outcomes such as school dropout, unemployment, and poor adult relationships [13] as well as social functioning issues [14].

Although irritability is not considered one of the core and definitive features of autism spectrum disorder (ASD), it is highly prevalent and predicts internalizing problems in individuals with ASD [15, 16]. Symptoms of irritability and emotional dysregulation are linked to significant daily impairment and social functioning difficulties in both ASD and non-ASD populations [14, 17]. Children with Rasopathies exhibit a high prevalence of social skills impairments compared to control groups [18]; investigating irritability in this population might provide important insights into the social functioning challenges associated with Rasopathies.

While irritability has not been extensively explored in Rasopathies [21, 22], it is particularly common and detrimental in children with neurodevelopmental disorders [8, 25–27], including neurogenetic syndromes, such as copy number variants (CNVs)[26, 28]. Moreover, evidence for the effective treatment of irritability is increasing [27]. A greater understanding of irritability and emotional dysregulation in different Rasopathies may promote the development of prevention strategies and more precise treatments in that population. Furthermore, this knowledge may give better insights into other key substantial impairments prevalent in Rasopathies, namely social skills impairments.

Therefore, this study aims to 1. Investigate whether children with Rasopathies are more irritable in comparison to TD and characterize the specific irritability phenotype of each Rasopathy group (NS and NF1); 2. Explore the association between irritability and behavioral disorders previously related to emotional dysregulation in Rasopathies and whether Rasopathy status influences this association. 3. Explore the association between irritability and social skills impairments in Rasopathies and whether Rasopathy status influences this association.

## Materials and Methods

### Participants

The study sample included a total of 174 children aged 4 to 17 years. The Rasopathies cohort included 113 children (mean age = 9.65, females = 64) and was constituted of 85 children with NS (mean age = 9.84, females = 51) and 28 children with NF1 (mean age = 9.09, females = 13). The age and sex-matched control cohort included 61 typically developing children (TD, mean age = 9.19, females = 34). Demographic characteristics of the study sample are shown in Table 1. Additional data about participants, recruitment, written consent, and exclusion criteria are shown in supplementary materials.

### Cognition

Cognition was assessed using the Wechsler Abbreviated Scale of Intelligence (WASI, [29], Wechsler Preschool and Primary Scale of Intelligence (WPPSI-III, [30], or Wechsler Intelligence Scale for Children (WISC-IV, [31]) depending on the participant’s age and in-person or remote status. The full-scale intelligence quotient (FSIQ) was selected as the global cognition measure.

### Behavioral Assessments

Both parents of children from all samples completed the Child Behavior Checklist (CBCL), an established and well-supported parent-report of behavioral and emotional problems in children aged 6–18 years of age [32] and the Social Responsiveness Scale (SRS) [33], a widely established parent-report assessment used in research and clinical settings in individuals between 4 and 18 years of age. The resulting scores from both parents were averaged to obtain individual scores for each participant. When a questionnaire was filled out by only one of the parents, that score was taken as the representative score for the child.

### Irritability

Consistent with previous research examining irritability in children [34, 35], irritability was assessed using a scale derived from the sum of three items from CBCL [32]. See Table 2 for irritability scale dimensions. Parents rated each statement regarding their child as follows: 0-the statement is not true, 1-the statement is somewhat or sometimes true, or 2-the statement is very true or often true. Higher scores indicate greater severity of symptoms (range 0–6).

### Emotional dysregulation-related disorders

To capture aspects of emotional dysregulation not covered by the irritability scale, we examined behavioral indices previously linked to emotional dysregulation [36, 37] and known to be prevalent in children with Rasopathies [19, 20, 38]: ADHD symptoms, defiance, and anxiety/depression symptoms.

We assessed ADHD symptoms using the t-score from the attention problems subscale of the CBCL [32] which also captures symptoms of dysregulation and impulsive behaviors related to ADHD [25, 39]. Consistent with previous studies [40–43], defiance was differentiated from irritability and was measured using a defiance scale based on the sum of three items from the CBCL [32] (range 0–6). The scale includes items of disobedience and disruptive behaviors, not part of the irritability scale. See Table 2. Consistent with previous research, anxiety/depression symptoms, internalizing symptoms linked to emotional dysregulation, [36, 44] were assessed using the t-score of the anxious/depressed subscale of the CBCL [32]. The broader CBCL internalizing score was not used in the study to avoid overlapping symptoms of irritability and social skills impairments.

### Social skills impairments

To measure social skills impairments in children with Rasopathies, we used the t-score of the total SRS scale [33], with higher total scores indicating greater severity of social impairment.

## Statistical analysis

Analysis was performed with R software, version 4.4.0. To compare differences in group demographic and cognitive variables (age, FSIQ, sex, and highest parent education), we conducted pairwise two-sample t-tests, chi-square test or Fisher’s exact test between Rasopathies vs. TD depending on type of data and data normality. We then performed a subsequential analysis comparing NS vs. TD, NF1 vs. TD, and NS vs. NF1 to understand specific differences between Rasopathies groups.

To investigate whether children with Rasopathies are more irritable than TD children and examine the specific irritability phenotype of each Rasopathy group, mean group differences in irritability were calculated between the group pairs using pairwise two-sample t-tests or Mann-Witney tests, depending on data normality. Analysis were calculated between Rasopathies vs. TD; subsequently, between NS vs. TD, NF1 vs. TD, and NS vs. NF1. The same subsequential steps were applied to calculate differences in emotional dysregulation indices, including ADHD symptoms, defiance symptoms, anxiety/depression symptoms, and social skills impairments. All p-values were corrected using the Bonferroni method reducing Type I errors (multiple comparisons). Associations between irritability, ADHD, defiance, anxiety/depression, and social skills impairments were assessed using Spearman’s correlation coefficient.

To assess how Rasopathy status influences the association between irritability and other emotional dysregulation indices, we employed a multiple linear regression model for each emotional dysregulation-related disorder (ADHD, defiance, and anxiety/depression) as the outcome variable, with irritability, group, and the interaction between irritability and group as predictors. Age, sex, highest parent education, and FSIQ variables were controlled for in all models. Models were applied first to the entire dataset, with Rasopathies and TD as the group variables. Subsequently, the models were applied to NS and TD, NF1 and TD, and NS and NF1 groups to explre specific differences between Rasopathies groups.

To assess how Rasopathy status influences the association between irritability and social skills impairments, we applied similar multiple linear regression models as described above but with social skills impairments serving as the outcome variable. For more data regarding the statistical analysis and data standardization see supplementary materials.

## Results

### Children with Rasopathies show high irritability symptoms

**Figure 1:**
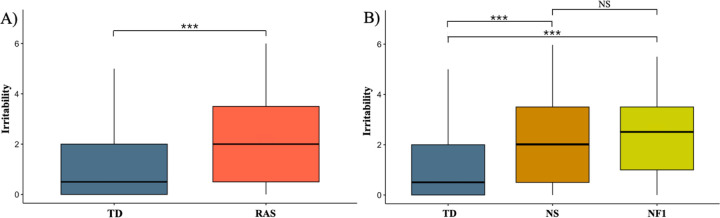
Irritability scores of children with Rasopathies compared to TD children A) Irritability scores of children with Rasopathies compared to TD children. B) Irritability scores comparisons between children with NS, NF1, and TD children. ***=p <0.001, ns=not significant. NF1=Neurofibromatosis type 1, NS=Noonan syndrome, RAS=Rasopathies, TD=Typically developing.

Children with Rasopathies showed higher irritability scores than TD children (mean difference = 1.09; 95% CI = 0.61, 1.57; p < 0.001). Both NS and NF1 groups scored higher in irritability compared to TD (NS vs. TD: mean difference = 1.07; 95% CI = 0.47, 1.68; p < 0.001; NF1 vs. TD: mean difference = 1.12; 95% CI = 0.30, 1.95; p = 0.004). No significant differences were found between NS and NF1 groups (mean difference = 0.05; 95% CI=−0.74, 0.84; *p* = 0.98). See Fig. 1. Descriptive statistics on all behavioral variables are reported in Table S1 (supplementary material).

Irritability is associated with emotional dysregulation-related disorders in Rasopathies with variable influences of NS and NF1 status.

**Figure 2:**
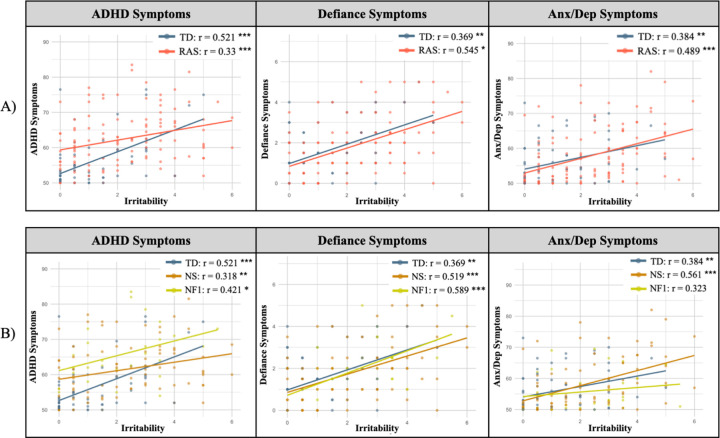
Association Between Emotional Dysregulation-related Indices and Irritability in Rasopathies A) Correlations between irritability and emotional dysregulation-related disorders including ADHD, defiance, Anx/Dep symptoms in Rasopathies and TD groups. B) Correlations between irritability and emotional dysregulation-related disorders including ADHD, defiance, Anx/Dep symptoms in NS, NF1, and TD groups. ADHD=attention deficit hyperactivity disorder symptoms, Anx/Dep= anxiety/depression symptoms, NF1=Neurofibromatosis type 1, NS=Noonan syndrome, Ras=Rasopathies, TD=typically developing.

As an initial step, we calculated the correlations between irritability and emotional dysregulation indices in the Rasopathies and TD groups and subsequently in the NS, NF1, and TD groups. See Fig. 2.

To explore if the Rasopathy status influenced the association between irritability and other emotional dysregulation indices, we employed multiple linear regression models, with ADHD, defiance, or anxiety/depression symptoms serving as the outcome variable in each model. The models were first applied to the entire dataset, with Rasopathies as the group variable, and subsequently applied to NS and TD, NF1 and TD, and NS and NF1 groups.

#### ADHD symptoms

We found a significant interaction between irritability and group in the NS and TD model (*p* = .032, η_p_^2^ = .03). The NS status influenced the association between irritability and ADHD symptoms, showing a weaker association in children with NS than in TD children. No interaction was found in the other models (Ras and TD, NF1 and TD, and NS and NF1). We found significant effects for irritability and group in the Ras and TD model (p < .001, ηp2 = 0.15; p = .009, ηp2 = .04), the NF1 and TD model (p < .001, ηp2 = 0.22; p < .001, ηp2 = .16) and NS and NF1 model (p = .002, ηp2 = 0.09; p = .013, ηp2 = .06).

ADHD symptoms were higher in children with irritability and in those with Rasopathies or NF1 compared to TD. See Table S1 for full results (supplementary material).

#### Defiance

We found no interaction between irritability and group in any of the models (Ras and TD, NS and TD, NF1 and TD, and NS and NF1). The Rasopathies status did not influence the association between irritability and defiance. We found an effect for irritability (p < .001, ηp2 = 0.21) with no effect for the group in the Rasopathies and TD model; defiance was higher in children with irritability, independently of the group (Rasopathies and TD). Similar results were found in all subsequent models (NS vs. TD, NF1 vs. TD, and NS vs. NF1). See Table S2 (supplementary material).

#### Anxiety/depression symptoms

We found no interaction between irritability and group in any of the models (Ras and TD, NS and TD, and NS and NF1), indicating that Rasopathies, NS, and NF1 status did not influence the association between irritability and anxiety/depression symptoms. Anxiety/depression symptoms were higher in children with irritability independently of the group, Ras and TD (p < .001, ηp2 = .12), NS and TD (p < .001, ηp2 = .16) and NS and NF1 models (p < .001, ηp2 = .10). Anxiety/depression symptoms were higher in females (Ras and TD (p < .021, ηp2 = .03), NS and TD (p < .025, ηp2 = .04)) and in older children (Ras and TD (p < .035, ηp2 = .03), NS and NF1 (p < .035, ηp2 = .04). The NF1 and TD model did not reach significance. See Table S3 (supplementary material).

#### Irritability is associated with Social Skills Impairments in Rasopathies, with variable influences of NS and NF1 status

**Figure 3:**
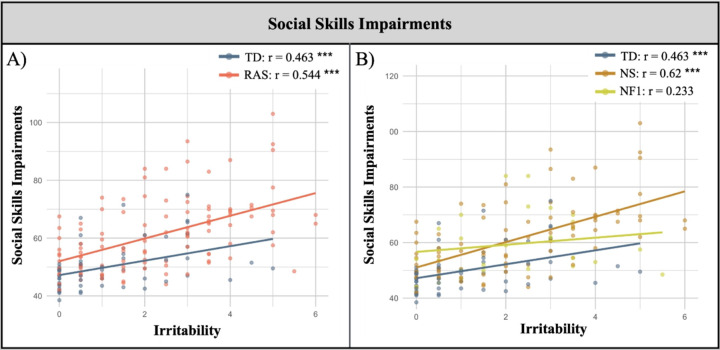
Associations of Irritability and Social Skills Impairments in Rasopathies A) Correlations with irritability and social skills impairments in the Rasopathies and TD groups. B) Correlations with irritability and social skills impairments in the NS, NF1, and TD groups. NF1=Neurofibromatosis type 1, NS=Noonan syndrome, TD=typically developing.

As an initial step, we calculated the correlations between irritability and social skills impairments first in the Rasopathies and TD groups and subsequently in the NS, NF1, and TD groups. See Fig. 3.

To explore to what extent the Rasopathy status influenced the association between irritability and social skills impairments, we employed multiple linear regression models, with social skills impairments serving as the outcome variable. We found a significant interaction between irritability and group in the NS and TD (*p* = .033, η_p_^2^ = .03), and the NS and NF1 models (*p* = .009, η_p_^2^ = .06) showing a stronger association between irritability and social impairments in NS compared to TD and NF1 children. No interaction was found between irritability and group in the Ras and TD, and NF1 and TD models.

We found a significant effect for irritability and group in the Ras and TD (p < .001, ηp2 = .17; p = .038, ηp2 = .03) and NF1 and TD models (p = .01, ηp2 = .08; p = .031, ηp2 = .06) and an effect for FSIQ in the Ras and TD, NS and TD and, NS and NF1 models. Social skills impairments were higher in children with irritability and in those children with Rasopathies and NF1 compared to TD children. Social skills impairments were higher in children with lower FSIQ in all models except for the NF1 and TD model. See Table S4 (supplementary material).

## Discussion

We present novel and clinically significant findings from a study investigating irritability in children with Rasopathies. We found that children with Rasopathies are significantly more irritable than TD children. While irritability scores were similar between the Rasopathies groups, we demonstrated different influences of NS and NF1 status on the associations between irritability and ADHD symptoms and social skills impairments. Specifically, compared to TD, children with NS showed a weaker association between irritability and ADHD symptoms, suggesting irritability and ADHD in NS as correlated dimensions, but each involving separated or additional underlying mechanisms. Conversely, children with NS showed a significantly stronger association between irritability and social skills impairments compared to both the TD and NF1 groups, indicating potentially shared neurobiological mechanisms between irritability and social skills impairments in NS.

Our results of high irritability in children with Rasopathies align with previous findings of high rates of irritability found in children with neurodevelopmental disorders and neurogenetic disorders [8, 25, 26, 28]. Few studies have investigated irritability in Rasopathies [21, 22]. One study focused on manic and depressive symptoms in children with NS [22] and found a high recurrence of emotional dysregulation and irritability, while another investigating personality profiles in children with NF1 [21] found NF1 children to be more irritable and more prone to overreact to frustrations compared to TD children. The high irritability levels found in children with Rasopathies in our study, along with the similar levels between NS and NF1, could imply an undiscovered association between irritability and the RAS-MAPK pathway dysregulation. Future mechanistic research, including other Rasopathies besides NS and NF1, is needed to support our hypothesis. Our findings, together with results from previous studies in CNVs [26], highlight the clinical importance of including assessments of irritability in children with Rasopathies and other neurogenetic syndromes and developing strategies for the treatment of irritability in those specific populations. Although the mechanisms of irritability and their biological links to Rasopathies have not yet been thoroughly examined, imaging findings in Rasopathies, specifically evidence of alterations in the striatum associated with NS [45]—a region known to be involved in emotional dysregulation—might suggest potential shared biological substrates between irritability and Rasopathies. Research investigating the genetic and brain correlates of irritability in children with Rasopathies would help support this hypothesis.

NS status, but not NF1 status, influenced the association between irritability and ADHD symptoms, with a weaker association in NS compared to TD. While there is extensive research showing correlations between irritability and ADHD symptoms [10, 25, 39], population studies prove that high irritability can also be prevalent in the absence of ADHD symptoms [46]. The weaker association between irritability and ADHD symptoms found in children with NS compared to TD, suggests that irritability and ADHD symptoms are correlated yet distinct behavioral dimensions, supporting one of the previous conceptual models proposed in the literature [25]. The pathophysiologic basis of this model is explained by studies showing different brain anomalies in individuals with ADHD and individuals with irritability. While individuals with ADHD show anomalies on fronto/striato/cerebellar circuits, the anomalies in individuals with irritability extend to limbic and paralimbic regions, including the amygdala, hippocampus, and parahippocampus [47, 48]. In addition, genetic studies show shared genes, but not all, between individuals with

ADHD alone and ADHD with emotion dysregulation [49, 50]. A recent study using a developmental genetic approach suggested the existence of different types of irritability, with only some genetically associated with ADHD [10]. While current evidence is insufficient to decisively favor one model of irritability and ADHD over the others [25], our results, together with previous research [10, 47, 48, 50], support the notion that irritability and ADHD are highly correlated but distinct dimensions in Rasopathies, particularly evident in children with NS. While there is growing behavioral, neurological, and cellular evidence implying a connection between Rasopathies and ADHD [19, 51, 52], the biological connection between irritability and Rasopathies is yet to be proven. Longitudinal studies examining irritability and ADHD in children with Rasopathies could serve as developmental genetic models to understand these associations better.

Irritability was strongly associated with defiance in all group models, with no influence of the Rasopathies status in the association. Previously conceptualized as unidimensional, ODD is increasingly recognized as heterogeneous and multidimensional, with most studies identifying two dimensions: irritability and defiance [53]. Despite ODD’s high prevalence in children (65% in clinical samples) [54], few studies have examined the prevalence of disruptive disorders in rare genetic syndromes [55, 56], and data concerning ODD, specifically defiance symptoms in Rasopathies, is even more sparse [19, 57]. This is the first study to examine irritability and defiance as separate dimensions of ODD in children with Rasopathies. While no differences in defiance symptoms were found between the Rasopathies and TD groups, irritability was significantly higher in Rasopathies compared to TD children. Our findings support the literature regarding irritability and defiance as correlated but complementary dimensions of the ODD diagnosis [53, 58], dimensions that are differentially associated with internalizing and externalizing problems [42, 53]. Our novel findings could point to a more irritable ODD phenotype in children with Rasopathies, which is known to be more associated with internalizing problems in adulthood [41]. Better-targeted prevention measures and treatments, with a focus on internalizing disorders, could be offered to children with Rasopathies presenting with ODD symptoms. More research investigating ODD and its different dimensions in Rasopathies is needed to confirm our results.

This study is among the first to examine the association between irritability and social skills impairments in children with Rasopathies. We found NS status to influence the association between irritability and social skills impairments, with this association being stronger in NS children compared to TD children and to children with NF1. These findings support several empirical works in ASD and non-ASD populations, highlighting that irritable and anger-prone children are at a greater risk of social maladjustments [59–61]. Furthermore, research has found that irritability and social problems also share neurobiological substrates [62–64]. Recent studies investigating predictors of social behavior focused on children with NS, NF1 and other Rasopathies found pragmatic language abilities, ADHD symptoms, and emotional symptoms (anxiety and mood) to be associated with social impairments [18, 65]. In contrast to our research, these studies [18, 65] did not assess irritability specifically and did not find differences in predicting social behaviors between children with NS and NF1 [18]. Our results provide specific novel findings suggesting differences in the mechanisms involved in the irritability and social processes in children with NS and NF1. Furthermore, these findings propose irritability as a potential predictor of social skills impairments in NS and a potential target for the development of prevention and treatment strategies for social skills impairments in Rasopathies. More research examining these different processes in distinct Rasopathies will be beneficial.

### Limitations and future directions:

Despite the several strengths presented in this study, there are some limitations that need to be addressed. First, the study’s cross-sectional nature limits the ability to infer causative or longitudinal effects between variables. Future longitudinal studies in children with Rasopathies could provide insights into whether irritability predicts specific behavioral problems and social skills impairments over time. Second, there is the potential for ascertainment bias. Children recruited in our study had a diagnosed genetic disorder and referral for genetic testing is often based on the presence of developmental delay. Including population-based cohorts might offer more generalizable information about the incidence of irritability in this population. Third, the sample size of our study, especially for NF1 (28 children). While the NF1 sample is relatively similar to other studies in NF1, larger samples could enhance the power of the results and help us understand the influence of other demographic and genetic variables. Lastly, all measures used were parent-reported, and no specific irritability measures were used. Although these measures are well established and frequently used in research [34, 35, 66], further studies should incorporate observational, self-report, and psychophysiological measures, along with other biological correlates.

## Conclusions

We present clinically significant and applicable findings from a study investigating irritability in children with Rasopathies. We found that children with Rasopathies are significantly more irritable than TD children. While irritability scores were similar between the Rasopathies groups, we demonstrated different influences of NS and NF1 status on the associations between irritability and ADHD symptoms and social skills impairments, potentially suggesting differences in the mechanisms underlying these processes. Moreover, the stronger association between irritability and social skills impairments in NS points to irritability as a potential target for developing prevention and treatment strategies for social skills impairments in NS.

## Supplementary Material

Supplement 1

## Figures and Tables

**Table 1:** Study Demographic Characteristics

	Ras (N = 113)	NS (N = 85)	NF1 (N = 28)	TD (N = 61)	Total (N = 174)	p values (Chi-Square or t-Test or Fisher’s Exact Test)			
						Ras:TD	NS:TD	NF1:TD	NS:NF1
Sex									
Female	64 (56.6%)	51 (60.0%)	13 (46.4%)	34 (55.7%)	98 (56.3%)	1.000	1.000	1.000	0.835
Male	49 (43.4%)	34 (40.0%)	15 (53.6%)	27 (44.3%)	76 (43.7%)				
Age									
Mean (SD)	9.65 (2.99)	9.84 (3.18)	9.09 (2.30)	9.19 (2.31)	9.49 (2.77)	1.000	0.640	1.000	0.725
Range	[5.20, 17.4]	[5.20, 17.4]	[5.69, 13.8]	[4.05, 16.5]	[4.05, 17.4]				
FSIQ									
Mean (SD)	96.5 (13.1)	96.0 (13.2)	98.0 (13.1)	111 (10.5)	102 (14.1)	<0.001***	<0.001***	<0.001***	1.000
Range	[63.0, 123]	[63.0, 123]	[73.0, 122]	[89.0, 134]	[63.0, 134]				
Highest Parent Education									
High School Degree	17 (15.0%)	13 (15.3%)	4 (14.3%)	5 (8.2%)	22 (12.6%)	0.050*	0.015*	1.000	0.887
College Degree	42 (37.2%)	35 (41.2%)	7 (25.0%)	12 (19.7%)	54 (31.0%)				
Graduate Degree	53 (46.9%)	36 (42.4%)	17 (60.7%)	42 (68.9%)	95 (54.6%)				
Missing	1 (0.9%)	1 (1.2%)	0 (0%)	2 (3.3%)	3 (1.7%)				
Mutations									
PTPN11	64 (56.6%)	64 (75.3%)			64 (36.8%)				
SOS1	14 (12.4%)	14 (16.5%)			14 (8.0%)				
NS-OTHER	7 (6.2%)	7 (8.2%)			7 (4.0%)				
NF1	28 (24.8%)		28 (100%)		28 (16.1%)				

FSIQ = full-scale intelligence quotient, NF1 = Neurofibromatosis type 1, NS = Noonan syndrome, NS-Other = all other NS mutations in the study besides PTPN11 and SOS1, including RAF1, RIT1, KRAS, and NSML-associated mutations, Ras = Rasopathies, TD = typically developing.

**Table 2:** Irritability and Defiance Scales Dimensions

Irritability		
CBCL Item	CBCL Item Definition	CBCL Question Reference
Temper tantrums	Discrete episodes of excessive temper, frustration or upset, manifested by shouting, crying or stamping, and involving violence or attempts at damage directed against people or property.	cbcl_q95
Stubborn, sullen, irritable	The child is generally more prone to feelings of irritability, often reacting negatively or with resistance under minor provocation.	cbcl_q86
Sudden changes in mood	The child is generally more prone to experiencing sudden changes in mood or feelings, often shifting quickly between different emotional states without clear triggers.	cbcl_q87
**Defiance**		
**CBCL Item**	**CBCL Item Definition**	**CBCL Question Reference**
Argues a lot	The child often engages in frequent verbal disputes or disagreements with others, challenging opinions or instructions persistently.	cbcl_q3
Disobedient at home	The child is generally more prone to being disobedient at home, often refusing to follow household rules or the instructions of family members.	cbcl_q22
Disobedient at school	The child is generally more prone to being disobedient at school, often disregarding rules, instructions, or authority figures while in the classroom or school environment.	cbcl_q23

CBCL = Child Behavior Checklist
