# Supplementary material for: Irritability in Children with Rasopathies, Insights into Emotional Dysregulation and Social Skills Impairments
Source: Res Sq. 2024 Dec 19:rs.3.rs-5428038. Preprint. [Version 1] doi: 10.21203/rs.3.rs-5428038/v1 (PMC11702823; doi:10.21203/rs.3.rs-5428038/v1)
Supplement: Supplement 1 [file nihpprs5428038v1-supplement-1.pdf]

Supplementary Files

This is a list of supplementary files associated with this preprint. Click to download.

- SupplementarymaterialECAP.docx
